# Batumi Raptor Count: autumn raptor migration count data from the Batumi bottleneck, Republic of Georgia

**DOI:** 10.3897/zookeys.836.29252

**Published:** 2019-04-08

**Authors:** Jasper Wehrmann, Folkert de Boer, Rafa Benjumea, Simon Cavaillès, Dries Engelen, Johannes Jansen, Brecht Verhelst, Wouter M.G. Vansteelant

**Affiliations:** 1 BRC Foundation, Dijkgraaf 35, 6721NJ Bennekom, The Netherlands BRC Foundation Bennekom Netherlands; 2 Zostera B.V., Halve Raak 30, 2771 AD Boskoop, The Netherlands Zostera B.V. Boskoop Netherlands; 3 BirdLife Europe and Central Asia, Avenue de la Toison d’Or 67 (2nd floor), 1060 Brussels, Belgium BirdLife Europe and Central Asia Brussels Belgium; 4 Computational and Theoretical Ecology, Institute for Biodiversity and Ecosystem Dynamics, University of Amsterdam, Amsterdam, The Netherlands University of Amsterdam Amsterdam Netherlands

**Keywords:** Black Sea flyway, birds of prey, bottleneck, citizen science

## Abstract

One of the most important geographical bottlenecks for migrating raptors in the east African-Palearctic migration system is situated between the easternmost tip of the Black Sea and the Lesser Caucasus, just north of Batumi, in the Republic of Georgia. Since 2008, citizen scientists of the Batumi Raptor Count (BRC) have monitored the autumn raptor passage daily from mid-August until mid-October, collecting also detailed information about the age and sex of focal species. The full BRC dataset was recently made available through the Global Biodiversity Information Facility (GBIF). Here we describe how count data were collected, managed, and processed for trend analysis over the past 10 years. This dataset offers a unique baseline for monitoring the state of migrant raptor populations in the east African-Palearctic flyway in the 21^st^ century. We discuss potential pitfalls for users and hope that the open access publication of our data will stimulate flyway-scale and continent-wide collaboration for raptor migration monitoring in the Old World.

## Introduction

Counting migrant birds at strategic geographical locations, such as coastlines and topographic leading lines, may be a cost-effective way of monitoring trends in the abundance of wide-ranging common species and the timing of their migration. Migration counts can even offer an alternative to breeding bird surveys for species that behave secretively or breed in remote parts of the world or inaccessible habitats. Thus, monitoring sites across the globe along major flyways for soaring birds were established in the 20^th^ century, and standardized migration counts have been used ever since to monitor migrant raptors across the globe (Bednarz et al. 1990; Kjellen and Roos 2000; Shirihai et al. 2000; McCarty and Bildstein 2005; Bensusan et al. 2007; Farmer et al. 2007; Filippi-Codaccioni et al. 2010; Martín et al. 2016). At the beginning of the 21^st^ century, new monitoring sites were established in important raptor migration flyways, such as those in Costa Rica and Panama along the Central American flyway (Porras-Peñaranda et al. 2004; Batista et al. 2005), Radar Hill and Khao Dinsor in Thailand (DeCandido et al. 2004, 2008), and Thoolakharka in Nepal (DeCandido 2013). In this paper, we describe raptor migration count data collected by the Batumi Raptor Count (BRC), a raptor migration count project which was established in 2008 along the eastern Black Sea coast in the Republic of Georgia.

Various naturalists have reported on the mass aggregation of migrant raptors along the eastern Black Sea flyway (Villkonskii 1897; Andrews et al. 1977; Magnin 1989; Van Maanen et al. 2001; Abuladze 2008, 2013). With this in mind, the BRC project mobilized an international team to conduct a two-month survey in the area in 2008 and 2009. This team included several dozen volunteers, of which most were experienced migration counters and bird watchers. These pilot counts revealed that the numbers of raptors converging in the Batumi bottleneck were much larger than previously estimated, making the eastern Black Sea flyway one of the most important flyways in the African-Palearctic migration system and including over 1% of the global population of 10 raptor species (Verhelst et al. 2011). It hosts, by far, the most concentrated autumn passage of European Honey-buzzard *Pernisapivorus* (Linnaeus, 1758), Montagu’s Harrier *Circuspygargus* (Linnaeus, 1758), Pallid Harrier *Circusmacrourus* (Gmelin, 1770), and Western Marsh-harrier *Circusaeruginosus* (Linnaeus, 1758) recorded anywhere in the world.

By 2011, BRC settled on a count protocol targeting those species for which migration counts are most likely to capture ecologically relevant population trends and, thus, likely to provide a useful monitoring instrument, which can be used for conservation purposes. Target species were selected based on (1) the relevance of the annual flight at Batumi relative to global population estimates (Verhelst et al. 2011), (2) species’ conservation status, and (3) species-specific flight behavior and timing, e.g. the ease with which species can be counted. For example, soaring migrants are relatively easy to count as they often pass in large streams, usually clearly visible from a vantage point and within a few 100 meters above the landscape. In contrast, many small raptors (e.g. Eurasian Sparrowhawk *Accipiternisus* Linnaeus, 1758) are difficult to count as they pass solitarily and low above ground and the forest canopy, especially under cloudy conditions.

Targeting specific species allows us to better standardize our count effort and to obtain better data quality. That is, we are able to record higher proportions of observations with accurate identification, age, sex, and other relevant information. Furthermore, we fine-tuned our count strategies between species to obtain the highest possible quality of count data under local conditions. In addition to raptors, we also decided to record European Roller *Coraciasgarrulus* (Linnaeus, 1758), European Turtle-dove *Streptopeliaturtur* (Linnaeus, 1758), White Stork *Ciconiaciconia* (Linnaeus, 1758), Black Stork *Ciconianigra* (Linnaeus, 1758), and Eurasian Crane *Grusgrus* (Linnaeus, 1758). Counting these species requires very little additional effort from counters, while the information collected may help to monitor the precarious conservation status of these species in the east African-Palearctic migration system.

In this paper we describe the BRC dataset, which for the occasion of the 10^th^ anniversary of the BRC was made publicly available through the Global Biodiversity Information Facility (GBIF) at https://www.gbif.org/dataset/d19c0287-15ee-45fd-b810-d30e8026a785. We describe our study site and the local flight paths and strategies of different species, elaborate on the count protocol used by our volunteer counters, and explain our data management strategy. We also discuss potential pitfalls for users and provide useful scripts (https://bitbucket.org/batumiraptorcount/gbif-data) to process count data for trend analyses. In our opinion, migration count data should always be published with open access to build a collaborative raptor migration monitoring network across all African-Palearctic flyways. More generally, we hope our dataset will reinforce the appreciation of the global importance of the eastern Black Sea flyway for migrant birds. Our transparent and open research approach should stimulate regional policymakers in particular to undertake urgent conservation actions regarding the still widespread practice of illegal raptor shooting along the Georgian Black Sea coast (Magnin 1989; Van Maanen et al. 2001; Abuladze et al. 2011; Jansen 2013; Sandor et al. 2017).

### The global importance of the Batumi bottleneck

Since 2008, 35 species of raptors have been recorded at Batumi and more than one million raptors have been counted annually since 2012. After the first two years of pilot counts in 2008 and 2009, we compared annual species totals with global breeding bird population estimates of BirdLife International to assess the global importance of the Batumi bottleneck (Verhelst et al. 2011). Count procedures were further improved based on field experience gained during pilot counts and since 2011 counts have been made with consistently high effort each year. Based on these high-quality counts, we can now estimate annual counts separately across age groups, and compare the most recent breeding bird population estimates of BirdLife International (2018) to counts of adult birds for several target species (Table 1).

**Table 1.** Reassessing the global importance of BRC migration counts for target species based on high-quality data 2011–2018. Counts after 2011 were higher for most of these species due to improved count locations and seasonal coverage. Thanks to a large ageing effort we can now estimate annual abundance of adult raptors from total annual species counts corrected for unidentified birds, and these exceed 1% of the estimated world breeding population estimate for eight species and one subspecies. Calculations of annual totals per species and age group are explained further in this manuscript.

Species	Average count			World population estimate ^(2)^	Percentage at Batumi (2011–2018)
	All individuals (2008–2009) ^(1)^	All individuals (2011–2018)	Adult individuals (2011–2018)		
European Honey-buzzard	453,344	530,568	499,493 ^(3,4)^	280,000–420,000	119–178
Steppe Buzzard	257,829	296,030 ^(5)^	–	4,000,000	7 ^(6)^
Black Kite	83,139	136,953	95,025 ^(3,4)^	1,000,000–2,499,999	4–10
Lesser Spotted Eagle	4,794	7,715	3,153	40,000–60,000	5–8
Greater Spotted Eagle ^(7)^	148	464	168	3,300–8,800	2–5
Steppe Eagle ^(7)^	332	568	62	50,000–75,000	< 0.1
Booted Eagle	4,144	6,475	4,983	149,000–188,000	3
Short-toed Snake-eagle	675	1,427	887	100,000–200,000	< 1
Western Marsh-harrier	4,218	6,489	2,575	500,000–999,999	< 1
Montagu’s Harrier	5,194	6,927	3,227	100,000–499,999	1–3
Pallid Harrier	1,652	1,491	376	18,000–30,000	1–2
Levant Sparrowhawk	4,668	3,508 ^(8)^	–	10,000–19,999	18–35 ^(6)^

^1^Verhelst et al. (2011), ^2^BirdLife International (2018), for Steppe Buzzard (BirdLife International 2004), ^3^ based on separate age protocol (see chapter “Ageing raptors”), ^4^ including unknown amount of immature birds, ^5^ counts since 2012, ^6^ based on all individuals, ^7^ minimum number, migration period not fully covered, ^8^ experimental counts 2014–2017.

This reassessment strengthens our belief that globally important numbers of adult raptors pass through the Batumi bottleneck every year. The fact our counts of European Honey-buzzards exceed the largest global breeding population estimates (Table 1) indicates that the population must be far larger than currently estimated, especially considering that 10,000s of individuals breeding in Western and Central Europe migrate via other flyways.

### Geography, weather, and flight paths of migrant raptors

**Figure 1.**
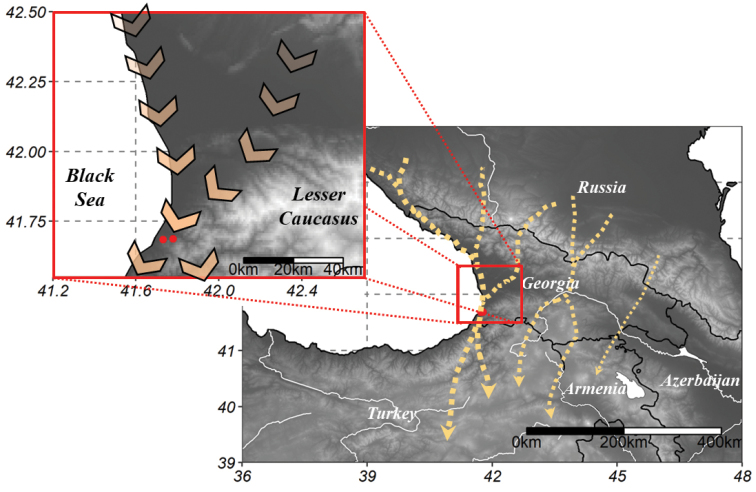
The Batumi bottleneck lies in the western coastal part of the trans-Caucasian migration corridor for soaring birds (main map, based on Abuladze 2013) and holds the strongest passage of migrant raptors at the eastern Black Sea flyway. The two hilltop count stations (red dots) are in the foothills of the Lesser Caucasus close to the city of Batumi in southwestern Georgia (inset).

Most raptors crossing the western half of the Greater Caucasus likely end up traveling across the Colchis Plains towards the Batumi bottleneck. The bottleneck is situated at the narrowest point between the eastern coast of the Black Sea and the foothills of the Lesser Caucasus, just to the north of the city of Batumi, in the Republic of Georgia (Fig. 1). Further inland, trans-Caucasian corridors are less well defined, but substantial numbers of raptors converge in large valleys and along watershed areas in both the Greater and Lesser Caucasus range, for example at Kazbegi (Tholin 2010), Mtkvari valley, Alazani, and the Javakheti Plateau (Abuladze 2013). In the eastern Caucasus an important bird migration flyway is situated along the west coast of the Caspian Sea in Azerbaijan, which is also used by substantial numbers of harriers and other raptors (Heiss and Gauger 2011). There is probably little exchange of soaring raptors between these trans-Caucasian migration corridors and the eastern Black Sea flyway, within or between years, although this needs to be confirmed empirically, for example through tracking studies.

There is no clear geographical boundary that limits the bottleneck on the eastern side, and anecdotal observations and satellite tracking data even suggest species like Steppe Buzzard *Buteobuteovulpinus* (Gloger, 1833) and Lesser Spotted Eagle *Clangapomarina* (Brehm, 1831) may pass as far as 50 km inland. However, on most days, and especially during the first half of the count season, a land-sea breeze circulation produces strong cloud cover over the mountains in the afternoon, causing the local flight paths of raptors to shift towards the coast during peak mid-day migration activity. In the second half of the season there is usually less cloud cover in the mountains, and the passage of late migrants like Steppe Buzzard and Lesser Spotted Eagle within the bottleneck becomes less concentrated along the coast (Vansteelant et al. 2014).

The passage of migrant raptors at Batumi is affected by weather conditions at a regional scale. Long periods of sustained rainfall, which we detected based on satellite images of cloud cover, cause an accumulation of soaring raptors to the north of Batumi (Wehrmann 2012). Therefore, such long periods of adverse weather tend to be followed by a short period of outstanding migration passage. From Batumi onwards, soaring birds continue further south along the coast or further inland using, for example, the Chorokhi valley into North East Turkey (Andrews et al. 1977).

## Count method

### Count station

Migration counts are performed simultaneously from two count stations to cover the approximately 12 km long transect line. The two count stations are located on hilltops with unobstructed view facing north into the landscape, and within visible range from each other. Station 1 (41°41.0683'N, 41°43.815'E) oversees the coastal migration from the village of Sakhalvasho and is situated 2.4 km from the coast and at 324 m above sea level. Station 2 (41°41.22'N, 41°46.7583'E) covers migration across the mountainous side of the bottleneck from the village of Shuamta and is situated 4 km to the east of Station 1 and at 414 m above sea level. During the beginning of the first pilot count from 17 August 2008 to the end of September 2008, Station 2 was located slightly further north in Davituri and at a lower elevation (Station 2A: 41°41.665'N, 41°47.3117'E) where visibility towards the east was considerably poorer than at the location that has been in use since October 2008.

**Figure 2.**
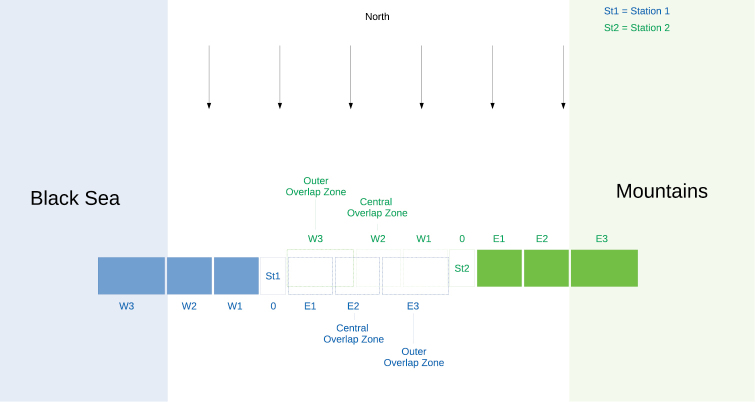
Schematic overview of distance and overlap zones of the two count stations at BRC showing the distance codes relative to the station from West3 (W3) to overhead (O) and East3 (E3).

Obviously, the use of two stations introduces the unwanted possibility for birds to be double counted (i.e. recorded by counters on both stations), especially when they pass between the counting stations. Count teams minimize such double counts by avoiding records in the outer overlap zone and using intensive radio communication between the stations for the central overlap zone (Fig. 2). All birds are recorded with a classification of their distance relative to the count station. Combined with time of passage this distance information can be used to detect and remove any remaining double counts during data processing. Figure 2 shows a schematic overview of the distance zones and the relevant overlap areas.

From 2008–2011, we occasionally used a third count station in the village of Chakvistavi (41°40.73'N, 41°52.0183'E), situated 7.5 km east of Station 2. These counts were conducted mainly during the European Honey-buzzard migration and yielded low numbers of migrants compared to the other two count stations. Taken together with the high cost of counting at this remote location and the methodical difficulties of increased double counts, we decided on a count strategy using two count stations.

### Count protocol

Information and experience gathered during the pilot counts in 2008–2010 guided the selection of target species for which we define priority and secondary species. For priority species, we expected long-term counts to generate relevant information for population monitoring. The selection of priority species is critical, because we determine our count season based on their phenology to ensure adequate monitoring.

In addition, we also target a number of secondary species that can easily be managed during counts of priority species because they pass in low numbers and are usually easily visible. These include enigmatic raptors like Short-toed Snake-eagle *Circaetusgallicus* (Gmelin, 1788) and Osprey *Pandionhaliaetus* (Linnaeus, 1758), threatened species like Egyptian Vulture *Neophronpercnopterus* (Linnaeus, 1758) and Saker Falcon *Falcocherrug* (Gray, 1834), and some enigmatic or threatened non-raptors like European Roller, pelicans *Pelecanus* spp. and storks. In contrast to priority species, however, we do not modify our count season in function of the phenology of these species.

Unfortunately, not all birds can be identified to species level in the field. Such birds are then categorized as accurately as possible into morphological groups (Table 2). To estimate the number of each priority species in each of these groups we need to account for all potentially confusing species (see full procedure under Pitfalls and recommendations). Consequently, the list of secondary species was extended with all species that are easily confused with priority species e.g. Hen Harrier *Circuscyaneus* (Linnaeus, 1766), Greater Spotted Eagle *Clangaclanga* (Pallas, 1811), and Steppe Eagle *Aquilanipalensis* (Hodgson, 1833).

**Table 2.** List of migratory species recorded at Batumi Raptor Count and the abbreviations used for each species. Column “status” highlights which species are considered as priority and secondary species. All other species are not counted in a standardized way and for some species the count was discontinued in 2011.

English name	Scientific name	Abbreviation	Status
**Buzzards**			
European Honey-buzzard	* Pernis apivorus *	HB	PRIORITY
Steppe Buzzard	* Buteo buteo vulpinus *	StepBuz	SECONDARY
Common Buzzard	* Buteo buteo *	CommonBuz	
Crested Honey-buzzard	* Pernis ptilorhynchus *	CrestedHB	
Rough-legged Buzzard	* Buteo lagopus *	RoughLB	
Long-legged Buzzard	* Buteo rufinus *	LongLB	
**Kites**			
Black Kite	* Milvus migrans *	BlackKite	PRIORITY
Red Kite	* Milvus milvus *	RedKite	
**Eagles**			
Lesser Spotted Eagle	* Clanga pomarina *	LesserSE	PRIORITY
Greater Spotted Eagle	* Clanga clanga *	GreaterSE	SECONDARY
Steppe Eagle	* Aquila nipalensis *	SteppeE	SECONDARY
Eastern Imperial Eagle	* Aquila heliaca *	ImperialE	SECONDARY
Golden Eagle	* Aquila chrysaetos *	GoldenE	
Booted Eagle	* Hieraaetus pennatus *	BootedE	PRIORITY
Short-toed Snake-eagle	* Circaetus gallicus *	ShortTE	SECONDARY
Osprey	* Pandion haliaetus *	Osprey	SECONDARY
White-tailed Sea-eagle	* Haliaeetus albicilla *	WhiteTE	
**Harriers**			
Western Marsh-harrier	* Circus aeruginosus *	Mar	PRIORITY
Montagu’s Harrier	* Circus pygargus *	Mon	PRIORITY
Pallid Harrier	* Circus macrourus *	Pal	PRIORITY
Hen Harrier	* Circus cyaneus *	Hen	SECONDARY
**Vultures**			
Egyptian Vulture	* Neophron percnopterus *	EgyptianV	SECONDARY
Griffon Vulture	* Gyps fulvus *	GriffonV	SECONDARY
Eurasian Black Vulture	* Aegypius monachus *	BlackV	SECONDARY
**Sparrowhawks and Goshawk**			
Levant Sparrowhawk	* Accipiter brevipes *	LevantSH	
Eurasian Sparrowhawk	* Accipiter nisus *	EurasianSH	
Northern Goshawk	* Accipiter gentilis *	Goshawk	
**Falcons**			
Lesser Kestrel	* Falco naumanni *	LesserKes	
Common Kestrel	* Falco tinnunculus *	CommonKes	
Red-footed Falcon	* Falco vespertinus *	RedFF	
Eleonora’s Falcon	* Falco eleonorae *	EleonoraF	
Merlin	* Falco columbarius *	Merlin	
Eurasian Hobby	* Falco subbuteo *	Hobby	
Lanner Falcon	* Falco biarmicus *	LannerF	SECONDARY
Saker Falcon	* Falco cherrug *	SakerF	SECONDARY
Peregrine Falcon	* Falco peregrinus *	Peregrine	SECONDARY
**Non-raptors**			
White Stork	* Ciconia ciconia *	WhiStork	SECONDARY
Black Stork	* Ciconia nigra *	BlaStork	SECONDARY
Eurasian Crane	* Grus grus *	EurasianCrane	SECONDARY
Demoiselle Crane	* Grus virgo *	DemCrane	SECONDARY
Great White Pelican	* Pelecanus onocrotalus *	WhiteP	SECONDARY
Dalmatian Pelican	* Pelecanus crispus *	DalmatianP	SECONDARY
European Roller	* Coracias garrulus *	Roller	SECONDARY
European Turtle-dove	* Streptopelia turtur *	TurtleD	SECONDARY
Common Wood-pigeon	* Columba palumbus *	WoodP	SECONDARY
Stock Dove	* Columba oenas *	StockD	SECONDARY
**Morphological groups**			
*Pernis* spp.		Pernis_SPEC	
*Buteo* spp. / *Pernis* spp.		Buzzard_SPEC	
Large Eagle		LargeEAGLE	
Montagu’s / Pallid / Hen Harrier		MonPalHen	
*Circus* spp.		Harrier_SPEC	
Eurasian / Levant Sparrowhawk		SparrowH_SPEC	
Sparrowhawk / Goshawk		SPH_Goshawk	
Large Falcon		LargeFALCON	
Hobby / Red-footed Falcon		Hobby_RedFF	
Common / Lesser Kestrel		Kestrel_SPEC	
*Falco* spp.		Falcon_SPEC	
Medium sized raptor		MediumRaptor	
Unknown raptor		Raptor_SPEC	
White / Black Stork		Stork_SPEC	

Targeting species entails that count-coordinators prioritize adequate counting of these species over others. For example, they ensure that sufficient people are counting flocks of priority and secondary species and scanning for birds even when a charismatic rarity passes by the station or when counters are distracted by outstanding passage of non-target species. However, users should be aware that following the analyses of pilot counts by Verhelst et al. (2011), we discontinued standardized surveys for some species in 2011.

### Duration of the count

We determined to organize daily counts from 17 August to 16 October based on the average phenology of the earliest and latest priority species during the counts of 2008–2010. More specifically, we start three days before the 1% quantile passage data of Montagu’s Harrier until three days after the 99% quantile passage date of Lesser Spotted Eagle only to be interrupted during thunderstorms or long spells of rain.

The daily count period starts at one hour after sunrise and ends two hours before sunset. These start and end times were based on (1) experience gathered during pilot counts, which showed that only a minor fraction of soaring birds pass outside this time window, (2) accessibility and transportation time to reach both count stations, and (3) the consideration that extending the count by an additional two hours imposes a heavy toll on the fitness of observers, especially in late August, when stations are occupied for up to twelve hours daily as it is.

However, with a growing network of local hosts and collaborators, we were eventually able to improve accessibility of and transportation to the count stations. We started conducting irregular experimental surveys at sunrise at Station 1 that revealed that a substantial proportion of daily harrier passage took place during early morning hours. Harriers can do this because they can rely on flapping-gliding flight during absence of favourable thermal conditions (Spaar and Bruderer 1997). Therefore, since 2015, we extend daily counts by counting from sunrise with minimum two counters on each station during main passage of Montagu’s and Pallid Harrier from 27 August to 27 September. Moreover, since 2015, irregular non-standardized counts are often conducted until sunset. We flagged the standardized and non-standardized observations in the column “filter” in our dataset. See Flagging standardized and non-standardized observations for further information on how to separate these data.

### Count coordination and data registry

Counts are conducted by experienced and less experienced bird watchers who volunteer to count at least for two weeks. Count coordinators screen, select, and schedule volunteers such that each station can be staffed by a team consisting of one coordinator and six to twelve counters, depending on migration intensity, diversity, and also counter availability. The team composition is equally balanced in terms of experience between both stations to be able to properly monitor the migration. The coordinator instructs the method of data recording, delegates tasks to the counting-team, communicates with the coordinator at the other station, and validates the data at the end of the day.

Migrants are recorded in the dataset within ten minutes after they have passed the transect line. For harriers, falcons, rollers, doves, and pigeons the data is recorded within five minutes. This avoids long uninterrupted counts, enabling us to study daily migration dynamic in more detail, and allows correcting for double counts between the two stations (see Data processing). Each record includes identification, distance zone relative to station, time, and for several species information on age, sex, and morph.

Note that in this way a single record can represent a solitary individual or an entire flock or stream, but also a subset of a larger flock or stream, as birds of different age, sex, or morph are recorded separately. At times of intense migration observations of solitary individuals passing a station in the same distance zone may be accumulated over several minutes and entered as a single record. Numbers associated with each record therefore do not represent group sizes.

### Alternative count strategy for European Honey-buzzard

European Honey-buzzard migration is characterized by its great intensity involving large streams of birds passing between both stations, sometimes going on uninterrupted for hours, which makes it hard for counters to separate streams from each other. We therefore count European Honey-buzzard predominantly from Station 1 during its main migration from 21 August to 9 September. During this period few other species mix with European Honey-buzzard in streams and good visibility at this time of the year usually allows us to count even the most inland streams from this station. Occasional records from Station 2 in the database were collected when poor visibility prohibited a single-station count. Any double counts resulting from this approach are eventually removed as for other species according to an automated procedure described later in this paper (see Double count removal).

### Identification of raptors using photography

Digital photography has become an important tool to aid identification of easily confused species such as adult and immature Greater Spotted or Steppe Eagle, adult Eastern Imperial Eagle *Aquilaheliaca* (Savigny, 1809), female Pallid Harrier, and Crested Honey-buzzard *Pernisptilorhynchus* (Temminck, 1821). Photography of difficult species has regularly been used for birds under poor visibility, or with inconclusive moult features.

### Ageing raptors

Most raptors can be aged and often sexed, based on morphological features (Forsman 2003, 2016). We collect age information for as many birds as possible. For the three most numerous species, European Honey-buzzard, Steppe Buzzard, and Black Kite *Milvusmigrans* (Boddaert, 1783), we sample daily age distributions for only a subset of birds.

*European Honey-buzzard, Black Kite, and Steppe Buzzard*

For European Honey-buzzard, Steppe Buzzard (only 2013), and Black Kite we use separate age protocols by sampling individuals rather than recording all birds as accurately as possible. These age protocols are restricted to birds passing between West1 (W1) to East1 (E1) of either count station. Second calendar year European Honey-buzzards are extremely rare in Europe and older immatures are impossible to distinguish from adults in the field. European Honey-buzzards overwinter in sub-Saharan Africa, and very few immatures come back to Europe with adults during their second calendar year (Corso 2012). In Batumi one record of a photographed second-calendar-year individual exists from autumn 2016 (Wright et al. in press). For immature Black Kites the determination of age in flight is challenging when large numbers pass the transect line in short time. We decided to monitor quantity rather than all age classes. Thus, for all three species, we only distinguish juvenile and non-juvenile birds. This data is recognizable by their abbreviations in the species column (HB_JUV, HB_NONJUV, BK_JUV, BK_NONJUV, SB_JUV, and SB_NONJUV).

*Harriers*

Ageing of female Harriers can be challenging and distant birds can be hard to distinguish from juvenile birds under poor visibility. Thus, we record birds as female colored, when they appear to be either in juvenile or female plumage. As it is often easier to determine age than to identify species for ringtail-harriers, we only trust records of Pallid and Montagu’s Harrier if they also include age information. Records without age information were therefore reclassified during data processing into the broader category “MonPalHen” (see Table 2).

*Large eagles*

To reliably distinguish large eagles such as Lesser Spotted Eagle, Greater Spotted Eagle, Steppe Eagle, and Eastern Imperial Eagle it is essential to first determine their age (Forsman 2003, 2016). We require age information for all large eagles for thorough identification. In contrast to harriers, however, we are not able to record every single Lesser Spotted Eagle observation with age information in the dataset on days with intensive migration, but we ensure on the station they have been correctly identified. Finally, subadult large eagles in their fifth calendar year can be hard to distinguish from older adults and are therefore always recorded as adult.

### Color morphs

*Booted Eagle*

Although the Booted Eagle *Hieraaetuspennatus* (Gmelin, 1788) occurs in pale, dark, and intermediate morphs in Batumi, we only separate pale individuals from all non-pale (here called dark) individuals (Forsman 2003, 2016). Dark morph individuals represent 51.9% of all Booted Eagles at Batumi (*n* = 42,475). This number matches with the known west-east gradient in the occurrence of dark morph Booted Eagle, with 20% dark morph individuals among eagles breeding in Spain and 80% among those breeding in eastern Russia (Karyakin 2007).

*Western Marsh-harrier*

A small number of Western Marsh-harriers are dark morph individuals (0.4%, *n* = 47,274). We only record males of the dark morph and note that substantial numbers of dark morph individuals may pass unnoticed when visibility or lighting is poor, or when they pass at far distance from either station, or when they are female colored.

### Experimental count of Levant Sparrowhawk and Red-footed Falcon

Although the migration pattern of Red-footed Falcon *Falcovespertinus* (Linnaeus, 1766) is interesting to monitor, and for Levant Sparrowhawk *Accipiterbrevipes* (Severtsov, 1850) pilot counts have confirmed globally relevant numbers in Batumi (Verhelst et al. 2011), we decided to do only an experimental count for these species, avoiding counting the potentially confusing species such as Eurasian Hobby *Falcosubbuteo* (Linnaeus, 1758) and Eurasian Sparrowhawk. The experimental survey was performed from 2014 to 2017. To avoid the very effortful count of predominantly solitary migrating Eurasian Sparrowhawk and Eurasian Hobby, Levant Sparrowhawks and Red-footed Falcons were counted only in flock sizes of five or more individuals, without determining age and sex. Methodical challenges resulting in low quality data made the data insufficient to monitor trends, and thus, we did not continue these counts.

### Steppe Buzzard

Following the pilot counts of 2008–2009 we initially decided not to monitor Steppe Buzzard at Batumi because we know large numbers of this species migrate over the interior of Georgia (Tholin 2010; Abuladze 2013) and because the migration of several priority species peaks during the Steppe Buzzard migration, which increases the counting effort. However, experience taught us that volunteer counters find it extremely demotivating not to count such a numerous migrant. Moreover, including this species formally in the count increases the possibility of reaching a million raptors per year. So, to help expand our international pool of potential volunteers, we resumed Steppe Buzzard counts in 2012.

### Data on illegal shooting

Because illegal shooting has a relevant impact on several migrant species in Batumi, we record data on health conditions as either injured or killed. This data helps to quantify illegal shooting per year and species (Jansen 2013; Sandor et al. 2017).

## Data management

**Figure 3.**
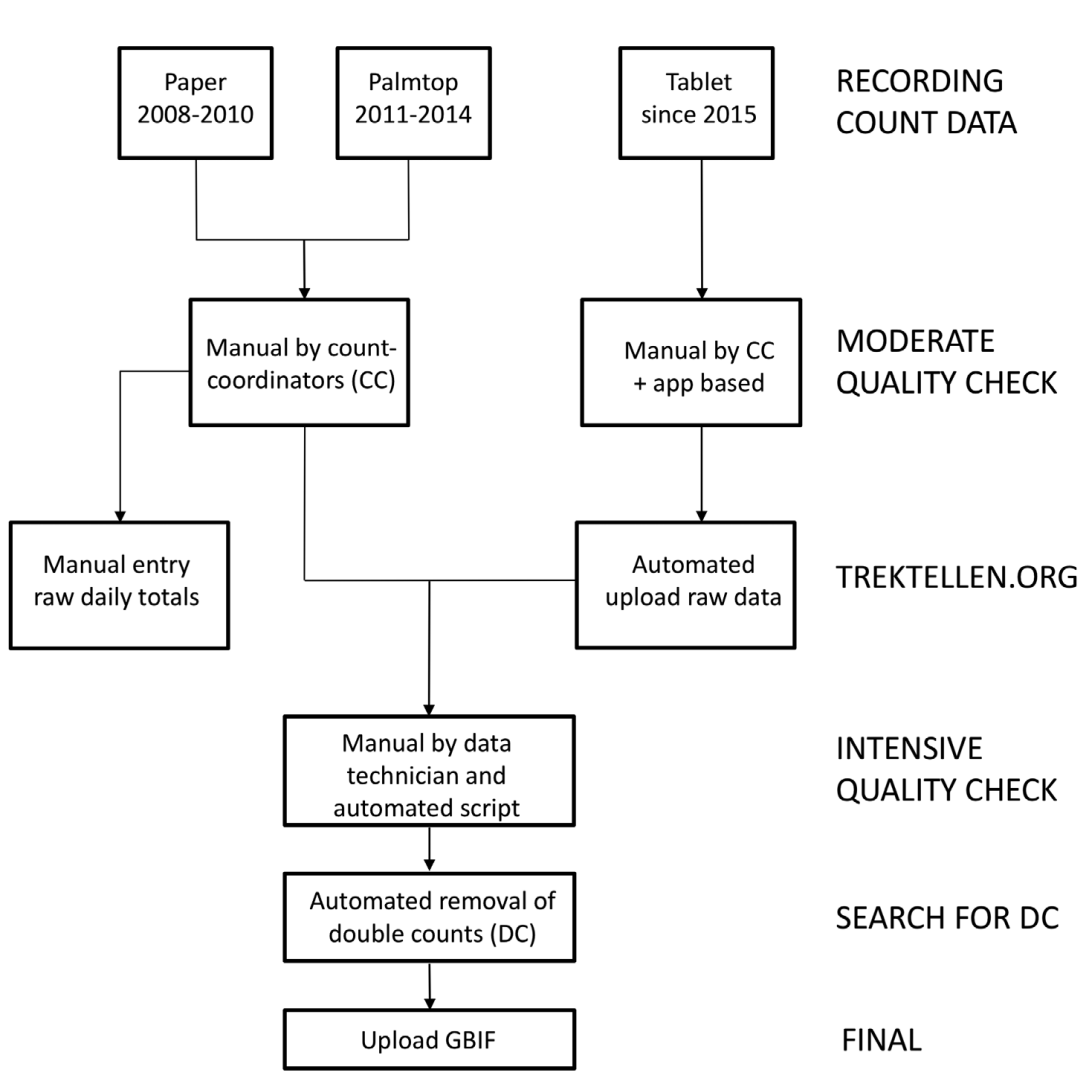
Diagram of data management and processing at BRC showing the data registry development from paper based entries in the beginning to the mobile application supported entries since 2015 with subsequent final data processing and upload to the GBIF database

The efficiency of data recording and management greatly improved over the years (Fig. 3). From 2008 to 2010 we took field notes on paper that afterwards had to go through the error prone and time consuming process of manual digitization. In 2011 we switched to handheld computers with a database entry module (palmtops), using paper notes in case of battery failure. In 2015, we changed to the mobile application of trektellen.org, which was partly developed based on the protocol and field experiences at Batumi Raptor Count.

During moderate quality check count-coordinators go quickly through the records to check for major errors (using simple criteria such as large flocks of rare birds, or specific age and sex combinations), and where possible correct them by discussing these observations with the relevant count team. Observations that are suspect due to insufficient information (e.g. a Greater Spotted Eagle cannot be identified without being aged) are degraded to a less specific level (in this example, to large eagle).

**Figure 4.**
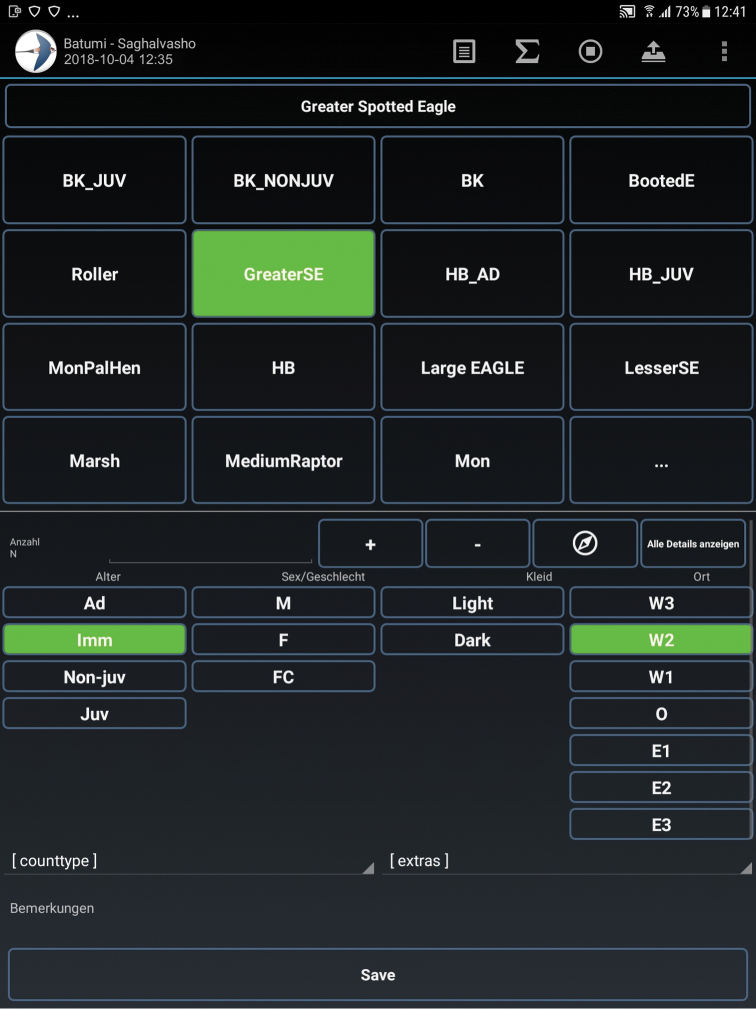
Screenshot of trektellen.org mobile application with the specific screen mode for raptor count at BRC.

From 2011 onwards, count data were downloaded daily from palmtops to a project computer. Daily totals were then computed and entered manually in the online database of trektellen.org to provide daily updates to the general public. Part of the moderate data quality check and the upload of count data to trektellen.org were finally automated in 2015 with the transition to the trektellen.org application. This application partly validates most of the records before entry by asking users for missing data in obligate fields or alerting users to incompatible information in species identification, sex, and age fields. An example of the main record-screen is shown in Figure 4.

## Data processing

### Intensive quality check

Regularly during the season assigned data technicians run an intensive search for errors in the dataset based on the same features that count-coordinators use for validation during the daily moderate quality check. As this process is very time consuming our experience has shown that count-coordinators usually do not identify all data errors during their daily routine. This process is completed by an automated script procedure that ensures final count protocol integrity and degrades records to a less specific level if still insufficient information is detected.

### Double count removal

An important final step in the production of the GBIF data product is to detect all remaining double counts in the overlapping count zones between the two stations. An automated procedure written in R detects potential double counts (on average 19,683 individuals annually) in different spatial and temporal windows for different species. In general, we distinguish between smaller and larger species because of differences in the distances at which they can be detected (spatial component) and differences in their flight behavior (temporal component). As a result, for smaller species such as falcons and harriers we consider double counts in a more narrow spatial overlap zone and a shorter time window than for larger species because the latter (e.g. eagles and buzzards) are visible from farther away. Soaring birds also take more time to pass through the bottleneck compared to actively flapping harriers, falcons, and sparrowhawks. To find the most suitable time frame in which double counts occur, we flagged known double counts in the field and found that most double counts are recorded within ± ten minutes at both stations in case of smaller and ± fifteen minutes in case of larger species.

We consider potential double counts not only within species, but also for corresponding higher orders of morphological groups. When a double count is detected, the observation with more details is kept. For example, three juvenile Pallid Harriers from Station 1 overlap with a record of eleven MonPalHen from Station 2, then the record of Station 1 has more details and is kept, while the record of Station 2 is subtracted by three. However, counters may flag records specifically as “single count” to exclude them of double count detection, if correctness is ensured through radio communication. On the other hand, a few recognized double counts are regularly and on purpose flagged as “double count” by observers to integrate them in the quality control of the detection script.

The double count approach is conservative, e.g. it detects more records as double counts than needed. As a result our data estimates the minimum number of individuals passing the bottleneck. However, double counts constitute less than 1% of all records, and so we do not expect double count removal to introduce any major bias in our data. For transparency the R-script for automated removal of double counts is available open access through the BitBucket account of the BRC (https://bitbucket.org/batumiraptorcount/gbif-data).

### Flagging standardized and non-standardized observations

The dataset includes observations since 2011 for counts with standardized high quality observations (dataset column “filter” = 1), standardized early-morning counts of harriers conducted since 2015 (dataset column “filter” = 2), non-standardized observations from outside the seasonal and daily count period since 2011, or from pilot counts in 2008–2010, or from experimental counts (dataset column “filter” = 0). For most research purposes it is advised to only use the standardized observations.

## Results

### Final GBIF product: BRC migration counts since 2008

The entire reviewed and processed dataset is published open access in GBIF at https://www.gbif.org/dataset/d19c0287-15ee-45fd-b810-d30e8026a785 and will be updated annually after each season. Table 3 provides detailed column descriptions of the final data product. The dataset up to 2018 includes over 400,000 observation records from standardized, pilot, experimental, and irregular counts. Table 4 provides processed year totals of all target species and their corresponding morphological groups since 2011 excluding non-standardized records. Table 5 shows the positive effect on total numbers of harriers since the recently integrated early morning counts have been started. The totals for all raptors (Table 6), including standardized and non-standardized species records, reach over one million raptors at the Batumi bottleneck every year during autumn migration.

**Table 3.** Detailed column description of the final GBIF product of BRC.

Column	Content
id	consecutive number
date	YYYY-MM-DD
time	hh:mm:ss
species	abbreviation of species or higher order of morphological groups (see Table 1)
number	processed number of migrants excluding double counts
north	number of migrants going north
station	number of the station, values: 1, 2
location	distance zone, values: W3, W2, W1, O, E1, E2, E3, E4
age	values: ad = adult, imm = immature, nonjuv = non-juvenile, juv = juvenile
sex	values: f = female, fc = female coloured (either juv or f), m = male
morph	values: dark, light, ful = fulvescens, mel = melanistic, leu = leucistic
health	health condition: kil = killed, inj = injured
remark	text with additional informations
dcremark	shows the corresponding record ID of the associate double count record with the concurrent number that is either subtracted (-) or kept (+)
filter	0 = non-standardized, 1 = standardized, 2 = standardized early morning count

**Table 4.** Year totals of all target species and their corresponding morphological groups based on standardized species records during autumn migration (dataset column “filter” = 1).

Species	2011	2012	2013	2014	2015	2016	2017	2018
European Honey-buzzard	370,587	643,212	427,183	656,171	559,790	509,112	518,242	485,917
Steppe Buzzard	–	185,317	415,439	486,467	199,620	203,210	281,403	300,757
Buzzard_SPEC	2,054	178	184	217	0	1,732	0	15,621
Black Kite	80,206	101,279	104,374	104,669	118,466	163,239	159,161	149,077
Lesser Spotted Eagle	5,844	4,536	3,845	2,467	4,088	3,721	4,696	4,278
Greater Spotted Eagle	220	160	203	243	397	273	331	426
Steppe Eagle	183	165	348	260	477	249	271	437
Eastern Imperial Eagle	37	11	37	46	33	29	44	62
Booted Eagle	6,497	7,001	6,150	6,143	6,639	7,370	6,814	5,188
Short-toed Eagle	1,293	1,348	1,376	1,405	1,329	1,443	1,436	1,788
Osprey	122	80	147	103	136	143	119	129
LargeEAGLE	2,646	2,390	6,446	7,256	2,404	1,967	3,417	5,343
Western Marsh-harrier	5,084	5,526	7,597	7,036	7,296	5,523	6,422	7,334
Montagu´s Harrier	2,753	5,010	3,245	2,802	2,997	2,506	2,967	1,794
Pallid Harrier	365	801	747	838	702	358	1,553	495
Hen Harrier	6	19	16	41	29	5	36	40
MonPalHen	4,314	6,650	5,398	5,892	5,103	2,994	2,212	4,907
Harrier_SPEC	32	10	30	15	6	33	22	38
Egyptian Vulture	40	24	36	19	27	28	19	17
Griffon Vulture	2	1	9	9	1	4	4	3
Eurasian Black Vulture	1	0	0	0	1	0	1	0
Lanner Falcon	1	0	0	0	0	0	0	0
Saker Falcon	2	1	1	3	2	1	0	1
Peregrine Falcon	31	48	28	32	40	26	26	22
LargeFALCON	14	11	5	1	6	1	1	8
MediumRaptor	9,774	14,722	160,557	32,124	53,303	45,299	36,231	92,104
Raptor_SPEC	0	0	0	0	0	3	277	428
White Stork	572	444	417	1,422	199	459	410	577
Black Stork	992	1,268	1,483	1,465	1,249	1,200	1,419	1,750
Stork_SPEC	0	10	25	0	0	0	0	14
Eurasian Crane	4	21	42	114	212	26	100	165
Demoiselle Crane	0	0	0	0	0	0	0	0
Great White Pelican	0	0	0	0	2	0	0	0
Dalmatian Pelican	0	0	3	0	0	2	0	0
European Roller	1,253	1,778	1,477	2,161	450	1,159	702	922
European Turtle-dove	–	–	4,571	1,099	461	1,934	1,714	686
Common Wood-pigeon	–	–	402	1,580	32	450	157	292
Stock Dove	–	–	1,300	764	800	765	387	713

**Table 5.** Year totals of harrier species based on standardized species records during autumn migration including standardized early morning counts since 2015 (dataset column “filter” = 1 and 2).

	Including early morning counts				Additional proportion (%) from early morning counts			
	2015	2016	2017	2018	2015	2016	2017	2018
Western Marsh-harrier	8,458	6,111	7,418	8,081	16	11	16	10
Montagu’s Harrier	3,262	2,781	3,591	1,938	9	11	21	8
Pallid Harrier	748	364	1,721	517	7	2	11	4
Hen Harrier	29	6	36	41	-	-	-	-
Mon / Pal / Hen Harrier	5,589	3,453	3,128	5,447	10	15	41	11
Harrier spp.	8	58	36	93	-	-	-	-
**Total**	**18,094**	**12,773**	**15,930**	**16,117**	**12**	**12**	**21**	**10**

**Table 6.** Year totals for raptors between 2008 and 2018 based on standardized (dataset column “filter” = 1 and 2) and non-standardized (dataset column “filter” = 0) species records. More than 1 million raptors have been registered during autumn migration in Batumi every year since 2012.

Dataset column filter	2008	2009	2010	2011	2012	2013	2014	2015	2016	2017	2018
1	-	-	-	492,118	978,528	1,143,468	1,314,297	962,947	949,305	1,025,735	1,076,244
2	-	-	-	-	-	-	-	1,961	1,354	2,718	1,509
0	881,838	917,119	504,825	1,739	31,428	56,294	36,500	65,761	92,939	26,031	53,767
**Total**	**881,838**	**917,119**	**504,825**	**493,857**	**1,009,956**	**1,199,762**	**1,350,797**	**1,030,669**	**1,043,598**	**1,054,484**	**1,131,520**

### Pitfalls and recommendations for analyses

Anyone using the GBIF data product is encouraged to contact the BRC team (research@batumiraptorcount.org) to discuss potential pitfalls for their specific usage. We here make some general recommendations for how to deal with known pitfalls that will commonly affect all researchers planning to use BRC count data.

Using data directly from trektellen.org is discouraged, as this only presents totals based on raw data excluding complete data checks and correction for double counts. The trektellen.org dataset includes non-standardized records such as irregular observations of non-target species, birds counted outside standardized count periods, and pilot or experimental counts. We strongly recommend to only use the standardized records in the GBIF dataset by filtering all records with value = 1 in the column “filter”, and following the recommendations below to calculate daily and annual totals for each species.

### Correct for unidentified birds (UID) in estimation of daily species totals

**Figure 5.**
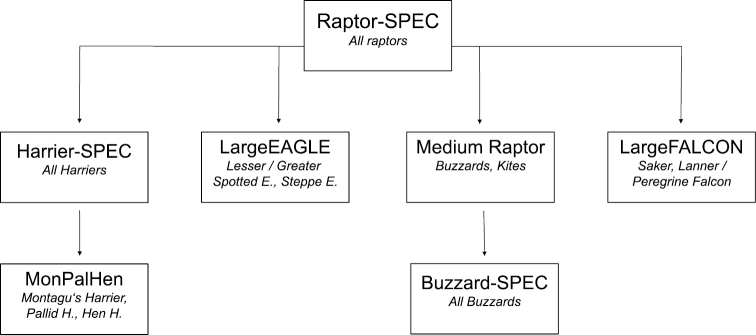
Hierarchy of morphological groups used to estimate how many “unidentified birds” belong to each species level shown only for target species at BRC.

Raptors can be difficult to identify especially when large streams of soaring migrants pass in the far east or west of the bottleneck or when visibility is poor. In such cases birds are more often identified to general morphological groups. In particular for ringtail-harriers, more birds are identified to the level of their morphological group (MonPalHen) than to actual species level. To include these unidentified birds in daily species totals for each higher morphological group, we estimate the daily proportion of the corresponding species, recalculate the daily total of that morphological group into species fractions, and add those to the daily totals of identified birds. We developed a script that iterates this procedure according to nested morphological groups used on the count stations (Fig. 5). For example, to estimate the daily total of European Honey-buzzard (HB), we must calculate how many of the birds recorded as Buzzard-SPEC, MediumRaptor, and Raptor-SPEC were likely to be HB. To do this, we first calculate the daily proportion of HB among all buzzards to estimate the number of HB in the category Buzzard-SPEC. We then add this number to the daily total of HB, recalculate proportion of HB among all buzzards and kites to estimate the number of HB in the category MediumRaptor. Finally, we estimate the daily proportion of HB among all raptors to estimate the number of HB in the category Raptor-SPEC.

An R script to estimate daily species totals (including and excluding UIDs) from the final GBIF data product described in this paper is made available through the BRC BitBucket account (https://bitbucket.org/batumiraptorcount/gbif-data). This script will read the GBIF dataset and produce a table with daily totals for each species. In case users run into difficulties with this script, the resulting table is available upon request by the BRC (research@batumiraptorcount.org).

### Using data about age, sex and morph

The number of birds for which detailed age and sex information can be recorded varies greatly, not only depending on intensity of migration, but also on experience and effort of volunteer observers. This also applies for European Honey-buzzards and Black Kites with their specific (separate) ageing protocol. Records with information on age, sex, or other plumage features, therefore, cannot be used directly to detect age-, sex-, or morph-specific migration strategies and trends. However, daily proportions of age groups, sexes, and/or morphs within the subset of accurately identified birds can be used to estimate daily totals of these groups from daily species totals (including the recalculation of morphological groups).

For European Honey-buzzards and Black Kites, we strongly recommend not estimating daily proportions of age groups and sexes from records in the regular count protocol (species values HB, BlackKite) but from the designated age protocol only (see Ageing raptors).

### Trend analyses

Data are likely not useful for trend analyses in species for which annual totals do not exceed 50–100 individuals (e.g. vultures, large falcons), species that have not been a target of the project, and species for which the count data does not cover the entire migration period (Greater Spotted Eagle, Steppe Eagle, Hen Harrier). Moreover, we strongly recommend analyzing standardized data only from 2011 onwards, when a fully-fledged count protocol was initiated.
